# On relativistic multipole moments of stationary space–times

**DOI:** 10.1098/rsos.180640

**Published:** 2018-07-18

**Authors:** Francisco Frutos-Alfaro, Michael Soffel

**Affiliations:** 1 School of Physics and Space Research Center of the University of Costa Rica, San Jose, Costa Rica; 2 Technical University Dresden and Lohrmann Observatory, Dresden, Germany

**Keywords:** quadrupole moment, stationary space–times, static metrics

## Abstract

Among the known exact solutions of Einstein's vacuum field equations the Manko–Novikov and the Quevedo–Mashhoon metrics might be suitable ones for the description of the exterior gravitational field of some real non-collapsed body. A new proposal to represent such exterior field is the stationary *q*-metric. In this contribution, we computed by means of the Fodor–Hoenselaers–Perjés formalism the lowest 10 relativistic multipole moments of these metrics. Corresponding moments were derived for the static vacuum solutions of Gutsunayev–Manko and Hernández–Martín. A direct comparison between the multipole moments of these non-isometric space–times is given.

## 1. Introduction

In 1968, Ernst [1,2] developed a complex procedure to simplify the Einstein field equations (EFE) for a stationary Weyl–Lewis–Papapetrou (WLP) type metric by means of two complex potentials. After this seminal work, several methods to find new solutions of the EFE were developed by Hoenselaers, Kinnersley and Xanthopoulos (HKX) [3] and Belinsky & Zakharov [4,5], among others. The HKX method was employed by Quevedo and Mashhoon (QM) to find new stationary solutions from the Erez–Rosen space–time as seed metric [6–8]. The Erez–Rosen metric is an exact vacuum solution of EFE representing a static metric with a mass and a quadrupole parameter (*M* and *Q*) [9]. The QM metric is an axially symmetric stationary vacuum solution with parameters *M*,  *a* and *q*_*n*_ with *n* being an (even) integer (*q*_2_ can be identified with a quadrupole parameter) [10].

The Manko–Novikov (MN) metric was derived by solving the Ernst equations using the techniques developed by Yamazaki [11,12], Cosgrove [13] and Dietz & Hoenselaers [14]. This metric has parameters *k*,  *α* (representing mass and rotation parameter, respectively) and *α*_*n*_ (*α*_2_ representing the quadrupole) [15]. Quevedo is proposing the stationary version of the *q*-metric to describe the gravitational field of compact stars [16–18]. Among the exact stationary vacuum solutions of EFE these metrics could be appropriate ones for the description of the exterior gravitational field of some real non-collapsed body simply because of their large freedom in choosing the multipole moments of the exterior gravitational field. Note, however, that the spin moments of higher order cannot be chosen freely but are totally determined by the set of parameters just mentioned; they might be far from those of a realistic body (e.g. [19,20]).

Geroch [21] and Hansen [22] (GH) have provided a definition for mass and spin multipole moments for a stationary space–time with asymptotic flatness. Simon & Beig [23] and Thorne [24] gave definitions of relativistic multipole moments even for non-stationary space–times. Gürsel [25] proved that the GH multipole moments are equivalent to the Thorne moments for stationary systems. From the Ernst formalism, Fodor, Hoenselaers and Perjés (FHP) [26] found an elegant method to derive explicit expressions for the multipole moments of a given stationary (axially symmetric) space–time with asymptotic flatness. Later, this method was generalized by Hoenselaers & Perjés [27]. Another method for deriving the relativistic multipole moments was devised by Ryan [28].

For most sufficiently massive astronomical bodies the shape (due to gravity and rotation) is close to that of an oblate spheroid, whose even mass-moments, in the Newtonian limit, are fully determined by the semi-major and minor axes. For an ordinary star the various multipole moments are not independent of each other but are determined by its mass, rate of rotation and the local equation of state. This also holds in the relativistic case, e.g. for neutron stars. Even for black holes, according to the no-hair conjecture all mass-moments are determined fully by the values for mass and angular momentum in the Kerr case. The situation where an object, for example, has no mass quadrupole but other higher mass multipole moments, is unphysical. This serves as motivation to analyse the multipole structure of these three space–times, namely the MN, QM and *q*-metrics.

This paper is organized as follows: the MN and the QM metrics are briefly reviewed in §2. The stationary version of the *q*-metric is introduced in this section too. We then derive the lowest relativistic multipole moments by means of the FHP method (in our notation $M_{2l}$ and $S_{2l+1}$ denote the GH mass and spin moments for the axially symmetric case) in §3. As limits the corresponding moments of the Schwarzschild, Kerr and Erez–Rosen metrics are contained. In §4, for three static cases, the Gutsunayev–Manko and Hernández–Martín (I and II) metrics, corresponding mass moments are rederived and compared with those from the MN, QM and *q*-metrics. We wrote a REDUCE program to get the first 11 multipole moments. Some conclusions are given in the last section.

## 2. The stationary metrics

The stationary space–time is represented by the WLP metric in prolate spheroidal coordinates (*t*,  *x*,  *y*, *ϕ*)

2.1 $$\begin{array}{rl} ds^{2} & =-f{\left(dt-\omega d\phi \right)}^{2}\\ & \;+\frac{\sigma^{2}}{f}\left[e^{2\gamma }(x^{2}-y^{2})\left(\frac{dx^{2}}{x^{2}-1}+\frac{dy^{2}}{1-y^{2}}\right)+(x^{2}-1)(1-y^{2})d\phi^{2}\right], \end{array}$$

where *σ* is a constant. This metric was transformed from cylindrical coordinates to these prolate coordinates which are defined by

2.2 $$\begin{array}{rl} \rho & =\sigma \sqrt{\left(x^{2}-1)(1-y^{2}\right)}\\ \;\mathrm{and}\;z & =\sigma xy. \end{array}\}$$

The functions *f* and *ω* are related to the twist scalar *Ω* through

2.3 $$f^{2}\nabla \omega =\rho \phi \times \nabla \Omega ,$$

where ***ϕ*** as the azimuthal unit vector and *ρ* the cylindrical coordinate.

The Ernst potential is E=f+iΩ, and the Ernst function is given by

2.4 $$\xi =\frac{1+E}{1-E}.$$

This Ernst function and its inverse *ξ*^−1^ fulfil the Ernst equation

2.5 $$(\xi \xi^{⋆}-1)\nabla^{2}\xi =2\xi^{⋆}{\left[\nabla \xi \right]}^{2}.$$

There are several techniques to solve this Ernst equation in these prolate coordinates; see for instance [3–5,12–14].

As examples, let us see what form the metric potentials (*f*,  *ω* and *γ*) take in these coordinates for the Kerr and Schwazschild metrics. The mapping from the Kerr spherical coordinates to the prolate coordinates is given by

2.6 $$\begin{array}{rl} \sigma x & =r-m\\ \;\mathrm{and}\;y & =\cos\theta , \end{array}\}$$

where

$$\sigma^{2}=m^{2}-a^{2}.$$

Using this mapping, the Kerr metric potentials take the following form:

2.7 $$\begin{array}{rl} f & =\frac{\sigma^{2}x^{2}+a^{2}y^{2}-m^{2}}{{\left(\sigma x+m\right)}^{2}+a^{2}y^{2}},\\ \omega & =2J\frac{\left(\sigma x+m)(1-y^{2}\right)}{\sigma^{2}x^{2}+a^{2}y^{2}-m^{2}}\\ \;\mathrm{and}\;e^{2\gamma } & =\frac{\sigma^{2}x^{2}+a^{2}y^{2}-m^{2}}{\sigma^{2}(x^{2}-y^{2})}, \end{array}\}$$

where *J* = *ma*.

For the Schwarzschild metric (set *a* = 0 in (2.7)) one has

2.8 $$\begin{array}{rl} f & =\frac{x-1}{x+1},\\ \omega & =0\\ \;\mathrm{and}\;e^{2\gamma } & =\frac{x^{2}-1}{x^{2}-y^{2}}, \end{array}\}$$

where *σ* = *m*.

### 2.1. The Manko–Novikov metric

This metric represents the space–time of a rotating massive object with mass and spin multipole moments. This metric has parameters: *k*, *α* and *α*_*n*_, *n* > 1. The parameters *k* and *α* are related to the mass and the rotation, respectively. It contains the following exterior metrics [15]:

— Schwarzschild, *α* = *α*_*n*_ = 0,
— Kerr, *α*_*n*_ = 0.

To compare it with the Kerr or QM metric one has to set

2.9 $$\begin{array}{rl} M & =k\left(\frac{1+\alpha^{2}}{1-\alpha^{2}}\right),\\ a & =-\frac{2k\alpha }{1-\alpha^{2}},\\ Q & =-k^{3}\alpha_{2}\\ \;\mathrm{and}\;k^{2} & =\sigma^{2}. \end{array}\}$$

If there is no rotation, *α* = 0, then *k* represents the mass. This solution has an infinite set of relativistic mass and spin multipoles. They correspond to the Kerr ones if *α*_*n*_ = 0.

The Ernst function is

2.10 $$\xi =e^{2\psi }\frac{A_{-}}{A_{+}},$$

where

2.11 $$A_{\mp }=x(1+\eta_{1}\eta_{2})+iy(\eta_{2}-\eta_{1})\mp (1-i\eta_{1})(1-i\eta_{2}).$$

From this Ernst function (2.10), employing methods developed by Yamazaki [12], Cosgrove [13] and Dietz & Hoenselaers [14], they generated the following metric potentials:

2.12 $$\begin{array}{rl} f & =\frac{A}{B}e^{2\psi },\\ \omega & =2k\frac{C}{A}e^{-2\psi }-4k\frac{\alpha }{1-\alpha^{2}}\\ \;\mathrm{and}\;e^{2\gamma } & =\frac{Ae^{2\chi }}{{\left(1-\alpha^{2}\right)}^{2}(x^{2}-1)}, \end{array}\}$$

where

2.13 $$\begin{array}{rl} A & =(x^{2}-1){\left(1+\eta_{1}\eta_{2}\right)}^{2}-(1-y^{2}){\left(\eta_{2}-\eta_{1}\right)}^{2},\\ B & ={\left[x+1+(x-1)\eta_{1}\eta_{2}\right]}^{2}+{\left[(1+y)\eta_{1}+(1-y)\eta_{2}\right]}^{2},\\ C & =(x^{2}-1)(1+\eta_{1}\eta_{2})[\eta_{2}-\eta_{1}-y(\eta_{1}+\eta_{2})]\\ & \;+(1-y^{2})(\eta_{2}-\eta_{1})[1+\eta_{1}\eta_{2}+x(1-\eta_{1}\eta_{2})]\\ \;\mathrm{and}\;\psi & =\sum_{n=1}^{\infty }\alpha_{n}\frac{P_{n}}{R^{n+1}}. \end{array}\}$$

The functions *η*_1_,  *η*_2_,  *χ*_1_ and *χ*_2_ are

2.14 $$\begin{array}{rl} \eta_{1} & =-\alpha e^{-2\chi_{1}},\\ \eta_{2} & =\alpha e^{2\chi_{2}},\\ \chi_{1} & =\sum_{m=1}^{\infty }\sum_{n=0}^{m}\alpha_{m}\left[(x-y)\frac{P_{n}}{R^{n+1}}-1\right]\\ \;\mathrm{and}\;\chi_{2} & =\sum_{m=1}^{\infty }\sum_{n=0}^{m}\alpha_{m}\left[{\left(-1\right)}^{m-n+1}(x+y)\frac{P_{n}}{R^{n+1}}+{\left(-1\right)}^{n}\right], \end{array}\}$$

with the following definitions:

2.15 $$\begin{array}{rl} R & =\sqrt{x^{2}+y^{2}-1}\\ \;\mathrm{and}\;P_{n} & =P_{n}\left(\frac{xy}{R}\right). \end{array}\}$$

The *γ* function is not involved in the calculation of the relativistic multipole moments. The interested reader may consult the references. In §3, the Ernst function *ξ* will be employed to determine the mass and spin multipole moments of the MN space–time.

### 2.2. The Quevedo–Mashhoon metric

The QM metric is a stationary axisymmetric solution of the vacuum EFE. This space–time has parameters: *M*, *a* and *q*_*n*_, *n* > 1, which represent the mass, the rotation parameter and additional multipole moment parameters of the object. It has another parameter, the Zipoy–Voorhees parameter, which we set *δ* = 1. In general, it presents a naked singularity. It contains the following exterior metrics [6–8,29]:

— Schwarzschild, *a* = *q*_*n*_ = 0,
— Erez–Rosen (ER), *a* = 0, *q* = *q*_2_, *q*_*n*_ = 0, (*n* > 2) [9],
— Kerr, *q*_*n*_ = 0,
— Hartle–Thorne (HT), *a* and *q* small, and *q*_*n*_ = 0, (*n* > 2) [10].

In our notation the parameters *m*≡*GM*/*c*^2^ and *a* have the dimension of a length and the parameters *q*_*n*_ are dimensionless.

This solution is characterized by an infinite set of relativistic mass and spin multipoles. They correspond to the Kerr ones if *q*_*n*_ = 0, and to the ER ones if *a* = 0 [8]. Boshkayev *et al.* [10] have shown how to get the HT from the QM metric. The HT metric is an approximate solution of the EFE for an object with three parameters, mass, spin and quadrupole moment. Although this solution is intended for slow rotation, it also has been employed to model fast rotating neutron stars. It has been argued (e.g. Boshkayev *et al.* [10]) that the QM space–time could be applied to model real astrophysical objects, such as neutron stars.

From two HKX transformations on the static Ernst potential $E_{0}=e^{-2\psi }$ for the ER space–time as seed metric, QM found the new Ernst potential, which is given by [6,8]

2.16 $$\xi =\frac{(a_{+}+ib_{+})+(a_{-}+ib_{-})e^{-2\psi }}{(a_{+}+ib_{+})-(a_{-}+ib_{-})e^{-2\psi }}.$$

From the Ernst function (2.16), they found the following functions [6–8,10]:

2.17 $$\begin{array}{rl} f & =\frac{A}{B}e^{-2\psi },\\ \omega & =-2\left(a+\sigma \frac{C}{A}e^{2\psi }\right)\\ \;\mathrm{and}\;e^{2\gamma } & =\frac{1}{4}{\left(1+\frac{m}{\sigma }\right)}^{2}\frac{A}{\left(x^{2}-1\right)}e^{2\chi }. \end{array}\}$$

The functions A, B and C are given by

2.18 $$\begin{array}{rl} A & =a_{+}a_{-}+b_{+}b_{-},\\ B & =a_{+}^{2}+b_{+}^{2}\\ \;\mathrm{and}\;C & =\left[x(1-y^{2})(\lambda +\eta )a_{+}+y(x^{2}-1)(1-\lambda \eta )b_{+}\right], \end{array}\}$$

where

2.19 $$\begin{array}{rl} a_{\pm } & =x(1-\lambda \eta )\pm (1+\lambda \eta )\\ \;\mathrm{and}\;b_{\pm } & =y(\lambda +\eta )\mp (\lambda -\eta ), \end{array}\}$$

with

2.20 $$\begin{array}{rl} \lambda & =\alpha e^{2\delta_{+}},\\ \eta & =\alpha e^{2\delta_{-}}\\ \;\mathrm{and}\;\alpha a & =\sigma -m. \end{array}\}$$

For the QM metric the potentials *ψ* and *δ*_ ± _ are of the form [30,31]

2.21 $$\psi =\sum_{n=1}^{\infty }{\left(-1\right)}^{n}q_{n}P_{n}(y)Q_{n}(x)$$

and

2.22 $$\begin{array}{rl} \delta_{\pm } & =\sum_{n=1}^{\infty }{\left(-1\right)}^{n}q_{n}\left[{\left(\pm 1\right)}^{n}\left(\frac{1}{2}\ln\left[\frac{{\left(x\pm y\right)}^{2}}{x^{2}-1}\right]-Q_{1}(x)\right)+P_{n}(y)Q_{n-1}(x\right)\\ & \;+\sum_{k=1}^{n-1}{\left(\pm 1\right)}^{k}P_{n-k}(y)(Q_{n-k+1}(x)-Q_{n-k-1}(x))]. \end{array}$$

The functions *P*_*l*_(*y*) and *Q*_*l*_(*x*) are Legendre polynomials of the first and second kind, respectively. The general form of the potential *χ* was determined by Quevedo [8,32]. To find the relativistic multipole moments, one does not need this *χ* function. The interested reader may consult the references.

With the Computer Algebra System REDUCE [33], we were able to check the validity of the Ernst equation directly without employing HKX transformations. In the next section, the Ernst function *ξ* will be employed to determine the relativistic multipole moments of the QM space–time.

### 2.3. The stationary *q*-metric

The *q*-metric is a generalization of the Schwarzschild metric with quadrupole parameter. The static version in spherical coordinates is given by [17,34]

2.23 $$ds^{2}=-h^{1+q}+h^{-q}\left[{\left(1+\frac{m^{2}\sin^{2}\theta }{r^{2}h}\right)}^{-q(2+q)}\left(\frac{dr^{2}}{h}+r^{2}d\theta^{2}\right)+r^{2}\sin^{2}\theta d\phi^{2}\right],$$

where *h* = 1 − 2 m r^−1^. It was obtained from the Zipoy–Voorhees (ZV) transformation with *δ* = 1 + *q*. From the parameters *m* and *q*, the mass and the quadrupole moment of the object are given by *M*_0_ = (1 + *q*)*m* and *M*_2_ =  − *m*^3^*q*(1 + *q*)(2 + *q*)/3, respectively. It is the simplest static metric with mass and quadrupole parameters. The rotating version of this metric has the following Ernst potential in prolate spheroidal coordinates:

2.24 $$E={\left(\frac{x-1}{x+1}\right)}^{q}\left[\frac{x-1+{\left(x^{2}-1\right)}^{-q}d_{+}}{x+1+{\left(x^{2}-1\right)}^{-q}d_{-}}\right],$$

where

$$\begin{array}{rl} d_{\pm } & =-\alpha^{2}(x\pm 1)h_{+}h_{-}{\left(x^{2}-1\right)}^{-q}+i\alpha [y(h_{+}+h_{-})\pm (h_{+}-h_{-})]\\ \;\mathrm{and}\;\;h_{\pm } & ={\left(x\pm y\right)}^{2q}. \end{array}$$

The parameter *α* is related to the rotation parameter *a* by (2.20). The prolate spheroidal coordinates are linked to the spherical coordinates through

2.25 $$\begin{array}{rl} x & =\frac{r}{\sigma }-1\\ \;\mathrm{and}\;\;y & =\cos\theta . \end{array}\}$$

The rotating metric can be read off from the general QM metric with ZV parameter. The Papapetrou potentials are

2.26 $$\begin{array}{rl} f & =\frac{A}{B},\\ \omega & =-2\left(a+\sigma \frac{C}{A}\right)\\ \;\mathrm{and}\;e^{2\gamma } & =\frac{1}{4}{\left(1+\frac{M}{\sigma }\right)}^{2}\frac{A}{{\left(x^{2}-1\right)}^{1+q}}{\left[\frac{x^{2}-1}{x^{2}-y^{2}}\right]}^{{\left(1+q\right)}^{2}}, \end{array}\}$$

where

2.27 $$\begin{array}{rl} A & =a_{+}a_{-}+b_{+}b_{-},\\ B & =a_{+}^{2}+b_{+}^{2} \end{array}\}$$

and

2.28 $$C={\left(x+1\right)}^{q}[x(1-y^{2})(\lambda +\eta )a_{+}+y(x^{2}-1)(1-\lambda \eta )b_{+}],$$

with

2.29 $$\begin{array}{rl} a_{\pm } & ={\left(x\pm 1\right)}^{q}[x(1-\lambda \eta )\pm (1+\lambda \eta )]\\ \;\mathrm{and}\;\;b_{\pm } & ={\left(x\pm 1\right)}^{q}[y(\lambda +\eta )\mp (\lambda -\eta )] \end{array}\}$$

and

2.30 $$\begin{array}{rl} \lambda & =\alpha {\left(x^{2}-1\right)}^{-q}{\left(x+y\right)}^{2q}\\ \;\mathrm{and}\;\;\eta & =\alpha {\left(x^{2}-1\right)}^{-q}{\left(x-y\right)}^{2q}. \end{array}\}$$

## 3. Relativistic multipole moments

There are several methods to get the spin and quadrupole moments from a given metric [25,26,28]. In this section, we apply the FHP procedure to the QM metric. An excellent review of the FHP formalism was given by Filter [35] in his diploma thesis. Filter also found the $S_{11}$ component in the same way. The procedure to obtain the relativistic multipole moments is the following [26]:

(i) employ the inverse Ernst potential *ξ*^−1^,
(ii) set *y* = cos*θ* = 1, and σx→1/z into *ξ*^−1^,
(iii) expand in Taylor series of *z* the inverse Ernst potential, and finally,
(iv) use the FHP formulae [26].

To get the relativistic multipole moment, we wrote a REDUCE program with the latter recipe.

### 3.1. The Manko–Novikov multipole moments

Taking the first 10 even members of the *ψ* function

3.1 $$\psi =\sum_{n=1}^{n=10}\alpha_{n}\frac{P_{n}}{R^{n+1}},$$

and defining

3.2 $$\beta^{2}=1-\frac{a^{2}}{m^{2}},$$

we derive the lowest relativistic multipole moments for the MN metric

3.3 $$\begin{array}{rl} M_{0} & =M\\ S_{1} & =S=-Mac\\ M_{2} & =Q+Sa\\ S_{3} & =4aQ+Sa^{2}\\ M_{4} & =-Sa^{3}-\frac{4}{7}Q(7a^{2}+2m^{2})-k\alpha_{4}\beta^{4}m^{4}\\ S_{5} & =-Sa^{4}-\frac{2}{21}aQ(42a^{2}+31m^{2})-4k\alpha_{4}\beta^{4}m^{4}a\\ M_{6} & =Sa^{5}+Q\left(4a^{4}+\frac{22}{7}m^{2}a^{2}+\frac{19}{33}m^{4}+\frac{60}{77}k^{3}\alpha_{2}m\right)\\ & \;+\frac{1}{11}k\alpha_{4}(44a^{6}-71m^{2}a^{4}+10m^{4}a^{2}+17m^{6})-k\alpha_{6}\beta^{6}m^{6}\\ S_{7} & =Sa^{6}+Qa\left(4a^{4}+\frac{490}{143}m^{2}a^{2}+\frac{584}{429}m^{4}-\frac{128}{143}k^{3}\alpha_{2}m\right)\\ & \;+\frac{1}{143}k\alpha_{4}a\left(572a^{6}-482m^{2}a^{4}-752m^{4}a^{2}+662m^{6}\right)-4k\alpha_{6}\beta^{6}m^{6}a \end{array}$$

3.4 $$\begin{array}{rl} M_{8} & =-Sa^{7}-Q\left(4a^{6}+\frac{1486}{429}m^{2}a^{4}+\frac{664}{429}m^{4}a^{2}+\frac{34}{143}m^{6}-k^{3}\alpha_{2}\left(\frac{2368}{429}ma^{2}-\frac{4364}{3003}m^{3}-\frac{40}{143}k^{3}\alpha_{2}\right)\right)\\ & \;+k\alpha_{4}\left(-4a^{8}+\frac{458}{143}m^{2}a^{6}+\frac{48}{11}m^{4}a^{4}-\frac{334}{143}m^{6}a^{2}-\frac{16}{13}m^{8}-\frac{226}{143}k^{3}\alpha_{2}\beta^{4}m^{5}\right)\\ & \;+2k\alpha_{6}\left(m^{8}+5m^{2}a^{6}-3m^{4}a^{4}-m^{6}a^{2}-2a^{8}\right)-k\alpha_{8}\beta^{8}m^{8}\\ S_{9} & =-Sa^{8}-aQ(4a^{6}+\frac{8586}{2431}m^{2}a^{4}+\frac{4384}{2431}m^{4}a^{2}+\frac{1310}{2431}m^{6}\\ & \;-k^{3}\alpha_{2}\left(\frac{21806}{2431}ma^{2}+\frac{15870}{17017}m^{3}+\frac{51464}{7293}k^{3}\alpha_{2}\right))\\ & \;-k\alpha_{4}a\left(4a^{8}-\frac{6762}{2431}m^{2}a^{6}-\frac{7662}{2431}m^{4}a^{4}-\frac{3286}{2431}m^{6}a^{2}+\frac{726}{221}m^{8}+\frac{5010}{2431}\beta^{4}Qm^{5}\right)\\ & \;-\frac{1}{17}k\alpha_{6}a\left(68a^{8}-94m^{2}a^{6}-126m^{4}a^{4}+262m^{6}a^{2}-110m^{8}\right)-4k\alpha_{8}\beta^{8}m^{8}a\\ M_{10} & =Sa^{9}+Q(4a^{8}+\frac{9550}{2717}m^{2}a^{6}+\frac{5032}{2717}m^{4}a^{4}+\frac{30650}{46189}m^{6}a^{2}+\frac{371}{4199}m^{8}\\ & \;-k^{3}\alpha_{2}\left(\frac{606490}{46189}ma^{4}+\frac{6\;399608}{969969}m^{3}a^{2}-\frac{182530}{138567}m^{5}-k^{3}\alpha_{2}\left(\frac{459700}{323323}m^{2}-\frac{3246296}{138567}a^{2}\right)\right))\\ & \;+\frac{1}{46189}k\alpha_{4}(184756a^{10}-131702m^{2}a^{8}-122630m^{4}a^{6}-64624m^{6}a^{4}\\ & \;+100166m^{8}a^{2}+34034m^{10}+Q(564540ma^{6}-1296110m^{3}a^{4}+898600m^{5}a^{2}\\ & \;-167030m^{7}-k^{3}\alpha_{2}\left(39150a^{4}-78300m^{2}a^{2}+39150m^{4}\right)))\\ & \;+\frac{1}{46189}\alpha_{4}^{2}M(30870a^{10}-154350m^{2}a^{8}+308700m^{4}a^{6}-308700m^{6}a^{4}\\ & \;+154350m^{8}a^{2}-30870m^{10})+\frac{1}{323}k\alpha_{6}(1292a^{10}-1714m^{2}a^{8}-1924m^{4}a^{6}\\ & \;+3136m^{6}a^{4}-104m^{8}a^{2}-686m^{10}+Qm(566m^{6}+1698m^{2}a^{4}-1698m^{4}a^{2}-566a^{6}))\\ & \;+\frac{1}{19}k\alpha_{8}\left(76a^{10}-257m^{2}a^{8}+268m^{4}a^{6}-22m^{6}a^{4}-112m^{8}a^{2}+47m^{10}\right)-k\alpha_{10}\beta^{10}m^{10}. \end{array}$$

### 3.2. The Quevedo–Mashhoon multipole moments

In this case, the Ernst function take the following simple form:

3.5 $$\xi =\frac{(\sigma x+ia)\;\tanh\;\psi +m}{(\sigma x-ia)+m\;\tanh\;\psi }.$$

Taking the first 10 even members of the function *ψ*,

3.6 $$\psi =\sum_{n=1}^{n=10}q_{n}P_{n}Q_{n}\;(P_{n}(1)=1).$$

From the ER metric, the static massive quadrupole is given by *Q* = 2*qMm*^2^/15. From the Kerr metric, one infers that the spin-dipole *S* = *Mac*. Using these relations, and *β* as in (3.2), one can write the relativistic multipole moments as follows:

3.7 $$\begin{array}{rl} M_{0} & =M\\ S_{1} & =S=Mac\\ M_{2} & =-Sa+\beta^{3}Q\\ S_{3} & =-Sa^{2}+2a\beta^{3}Q\\ M_{4} & =Sa^{3}\left(1+\frac{8}{21}\beta q_{2}\right)-\frac{2}{7}\beta Q(m^{2}+9a^{2})+\frac{8}{315}\beta^{5}q_{4}Mm^{4}\\ S_{5} & =Sa^{4}\left(1+\frac{52}{105}\beta q_{2}\right)-\frac{2}{7}\beta Qa(2m^{2}+11a^{2})+\frac{16}{315}\beta^{5}q_{4}Sm^{4}\\ M_{6} & =-Sa^{5}\left(1+\frac{62}{105}\beta q_{2}-\frac{16}{1155}q_{2}^{2}\right)+\frac{1}{231}Q\left(24q_{2}m^{2}(3\beta^{2}a^{2}-m^{2})+\beta (773a^{4}+258m^{2}a^{2}-8m^{4})\right)\\ & \;-\frac{8}{3465}\beta q_{4}M\left((37a^{6}+2m^{6})+3m^{2}a^{2}(11m^{2}-24a^{2})\right)+\frac{16}{3003}\beta q_{6}M\left(m^{6}-a^{6}-3\beta^{2}m^{4}a^{2}\right)\\ S_{7} & =-Sa^{6}\left(1+\frac{24}{35}\beta q_{2}-\frac{712}{19305}q_{2}^{2}\right)+\frac{4}{9009}Qa\left(623q_{2}m^{2}(3\beta^{2}a^{2}-m^{2})+39\beta \left(205a^{4}+96m^{2}a^{2}-4m^{4}\right)\right)\\ & \;-\frac{32}{3465}\beta q_{4}S\left((13a^{6}+m^{6})+m^{2}a^{2}(11m^{2}-25a^{2})\right)+\frac{32}{3003}\beta q_{6}S\left(m^{6}-a^{6}-3\beta^{2}m^{4}a^{2}\right)\\ M_{8} & =Sa^{7}(1+\frac{2656}{3465}\beta q_{2}-\frac{17632}{225225}q_{2}^{2}-\frac{64}{96525}\beta q_{2}^{3}\\ & \;+\frac{7088}{45045}\beta q_{4}-\frac{3616}{675675}q_{2}q_{4}+\frac{928}{45045}\beta q_{6}+\frac{128}{109395}\beta q_{8})\\ & \;+\frac{4}{3003}\beta Q\left(-2605a^{6}-1827m^{2}a^{4}+120m^{4}a^{2}-4m^{6}\right)\\ & \;+\frac{16}{45045}q_{2}Q\left(4922a^{6}-4848m^{2}a^{4}+1542m^{4}a^{2}+37m^{6}\right)\\ & \;+\frac{32}{6435}\beta q_{2}^{2}Q\left(4a^{6}-6m^{2}a^{4}+4m^{4}a^{2}-m^{6}\right)\\ & \;+\frac{16}{45045}q_{4}Q\left(452a^{6}-678m^{2}a^{4}+452m^{4}a^{2}-113m^{6}\right)\\ & \;+\frac{16}{45045}\beta q_{4}Mm^{2}\left(-830a^{6}+329m^{2}a^{4}+60m^{4}a^{2}-2m^{6}\right)\\ & \;+\frac{32}{45045}\beta q_{6}Mm^{2}\left(-86a^{6}+84m^{2}a^{4}-26m^{4}a^{2}-m^{6}\right)\\ & \;+\frac{128}{109395}\beta q_{8}Mm^{2}\left(-4a^{6}+6m^{2}a^{4}-4m^{4}a^{2}+m^{6}\right) \end{array}$$

3.8 $$\begin{array}{rl} S_{9} & =Sa^{8}(1+\frac{2936}{3465}\beta q_{2}-\frac{171568}{1276275}q_{2}^{2}-\frac{5504}{4922775}\beta q_{2}^{3}\\ & \;-\frac{5856}{425425}q_{2}q_{4}+\frac{8768}{45045}\beta q_{4}+\frac{1376}{45045}\beta q_{6}+\frac{256}{109395}\beta q_{8})\\ & \;+\frac{4}{3003}\beta Qa\left(-2545a^{6}-2406m^{2}a^{4}+188m^{4}a^{2}-8m^{6}\right)\\ & \;+\frac{8}{85085}Qq_{2}a\left(31842a^{6}-31188m^{2}a^{4}+9742m^{4}a^{2}+327m^{6}\right)\\ & \;+\frac{16}{328185}\beta q_{2}^{2}Qa\left(688a^{6}-1032m^{2}a^{4}+688m^{4}a^{2}-172m^{6}\right)\\ & \;+\frac{16}{85085}q_{4}Qa\left(2196a^{6}-3294m^{2}a^{4}+2196m^{4}a^{2}-549m^{6}\right)\\ & \;+\frac{32}{45045}\beta q_{4}Sm^{2}\left(-505a^{6}+186m^{2}a^{4}+47m^{4}a^{2}-2m^{6}\right)\\ & \;+\frac{32}{45045}\beta q_{6}Sm^{2}\left(-127a^{6}+123m^{2}a^{4}-37m^{4}a^{2}-2m^{6}\right)\\ & \;+\frac{256}{109395}\beta q_{8}Sm^{2}\left(-4a^{6}+6m^{2}a^{4}-4m^{4}a^{2}+m^{6}\right)\\ M_{10} & =-Sa^{9}(1+\frac{41318}{45045}\beta q_{2}-\frac{78304}{373065}q_{2}^{2}-\frac{65216}{26189163}\beta q_{2}^{3}\\ & \;-\frac{51552}{1616615}q_{2}q_{4}-\frac{1856}{4849845}\beta q_{2}^{2}q_{4}-\frac{18112}{14549535}q_{2}q_{6}+\frac{10448}{45045}\beta q_{4}\\ & \;-\frac{896}{2078505}q_{4}^{2}+\frac{33472}{765765}\beta q_{6}+\frac{10624}{2078505}\beta q_{8}+\frac{256}{969969}\beta q_{10})\\ & \;+\frac{1}{969969}\beta Q\left(2995461a^{8}+4048532m^{2}a^{6}-397840m^{4}a^{4}+27600m^{6}a^{2}-896m^{8}\right)\\ & \;+\frac{16}{2909907}q_{2}Q\left(-843844a^{8}+812776m^{2}a^{6}-238254m^{4}a^{4}-17939m^{6}a^{2}+962m^{8}\right)\\ & \;+\frac{32}{8729721}\beta q_{2}^{2}Q\left(-17008a^{8}+17082m^{2}a^{6}-148m^{4}a^{4}-8393m^{6}a^{2}+3372m^{8}\right)\\ & \;+\frac{16}{2909907}q_{4}Q\left(-173614a^{8}+259486m^{2}a^{6}-171744m^{4}a^{4}+42001m^{6}a^{2}+374m^{8}\right)\\ & \;+\frac{928}{323323}\beta q_{2}q_{4}Q\left(-5a^{8}+10m^{2}a^{6}-10m^{4}a^{4}+5m^{6}a^{2}-m^{8}\right)\\ & \;+\frac{9056}{969969}q_{6}Q\left(-5a^{8}+10a^{6}m^{2}-10a^{4}m^{4}+5a^{2}m^{6}-m^{8}\right)\\ & \;+\frac{16}{14549535}\beta q_{4}Mm^{2}\left(378672a^{8}-121361m^{2}a^{6}-49730a^{4}m^{4}+3450m^{6}a^{2}-112m^{8}\right)\\ & \;+\frac{896}{2078505}q_{4}^{2}Mm^{2}\left(-5a^{8}+10m^{2}a^{6}-10m^{4}a^{4}+5m^{6}a^{2}-m^{8}\right)\\ & \;+\frac{32}{14549535}\beta q_{6}Mm^{2}\left(58065a^{8}-54895m^{2}a^{6}+15035m^{4}a^{4}+1725a^{2}m^{6}-56m^{8}\right)\\ & \;+\frac{128}{2078505}\beta q_{8}Mm^{2}\left(330a^{8}-490m^{2}a^{6}+320m^{4}a^{4}-75m^{6}a^{2}-2m^{8}\right)\\ & \;+\frac{256}{969969}\beta q_{10}Mm^{2}\left(5a^{8}-10a^{6}m^{2}+10a^{4}m^{4}-5a^{2}m^{6}+m^{8}\right). \end{array}$$

### 3.3. The *q*-metric multipole moments

For this metric, the relativistic multipole moments are (*S*_0_ = *Mac*)

3.9 $$\begin{array}{rl} M_{0} & =M\left(1+q\frac{\sigma }{m}\right)\\ S_{1} & =S=S_{0}\left(1+2q\frac{\sigma }{m}\right)\\ M_{2} & =M\left(-m^{2}-3mq\sigma -q^{2}\sigma^{2}+\sigma^{2}-\frac{1}{3}q^{3}\frac{\sigma^{3}}{m}+\frac{7}{3}q\frac{\sigma^{3}}{m}\right)\\ S_{3} & =S_{0}\left(3a^{2}q^{2}-a^{2}+\frac{2}{3}a^{2}q^{3}\frac{\sigma }{m}-\frac{8}{3}a^{2}q\frac{\sigma }{m}-3m^{2}q^{2}-\frac{2}{3}mq^{3}\sigma -\frac{4}{3}mq\sigma \right)\\ M_{4} & =M(m^{4}+5m^{3}q\sigma +6m^{2}q^{2}\sigma^{2}-2m^{2}\sigma^{2}+\frac{46}{21}mq^{3}\sigma^{3}-\frac{166}{21}mq\sigma^{3}+\frac{19}{21}q^{4}\sigma^{4}\\ & \;-\frac{16}{3}q^{2}\sigma^{4}+\sigma^{4}+\frac{19}{105}q^{5}\frac{\sigma^{5}}{m}-\frac{16}{21}q^{3}\frac{\sigma^{5}}{m}+\frac{43}{15}q\frac{\sigma^{5}}{m})\\ S_{5} & =S_{0}(\frac{145}{63}a^{4}q^{4}-\frac{520}{63}a^{4}q^{2}+a^{4}+\frac{74}{315}a^{4}q^{5}\frac{\sigma }{m}-\frac{52}{63}a^{4}q^{3}\frac{\sigma }{m}+\frac{46}{15}a^{4}q\frac{\sigma }{m}\\ & \;-\frac{290}{63}a^{2}m^{2}q^{4}+\frac{410}{63}a^{2}m^{2}q^{2}-\frac{148}{315}a^{2}mq^{5}\sigma -\frac{256}{63}a^{2}mq^{3}\sigma +\frac{316}{105}a^{2}mq\sigma \\ & \;+\frac{145}{63}m^{4}q^{4}+\frac{110}{63}m^{4}q^{2}+\frac{74}{315}m^{3}q^{5}\sigma +\frac{44}{9}m^{3}q^{3}\sigma -\frac{8}{105}m^{3}q\sigma ) \end{array}$$

3.10 $$\begin{array}{rl} M_{6} & =M(-m^{6}-7m^{5}q\sigma -15m^{4}q^{2}\sigma^{2}+3m^{4}\sigma^{2}-\frac{775}{63}m^{3}q^{3}\sigma^{3}+\frac{1093}{63}m^{3}q\sigma^{3}\\ & \;-\frac{125}{21}m^{2}q^{4}\sigma^{4}+\frac{556}{21}m^{2}q^{2}\sigma^{4}-3m^{2}\sigma^{4}-\frac{11}{5}mq^{5}\sigma^{5}+\frac{8524}{693}mq^{3}\sigma^{5}\\ & \;-\frac{46997}{3465}mq\sigma^{5}-\frac{389}{495}q^{6}\sigma^{6}+\frac{127}{33}q^{4}\sigma^{6}-\frac{39397}{3465}q^{2}\sigma^{6}+\sigma^{6}-\frac{389}{3465}q^{7}\frac{\sigma^{7}}{m}\\ & \;+\frac{719}{3465}q^{5}\frac{\sigma^{7}}{m}-\frac{37}{55}q^{3}\frac{\sigma^{7}}{m}+\frac{337}{105}q\frac{\sigma^{7}}{m})\\ S_{7} & =S_{0}(\frac{27073}{19305}a^{6}q^{6}-\frac{21427}{3861}a^{6}q^{4}+\frac{287647}{19305}a^{6}q^{2}-a^{6}+\frac{2986}{45045}a^{6}q^{7}\frac{\sigma }{m}\\ & \;-\frac{316}{6435}a^{6}q^{5}\frac{\sigma }{m}+\frac{94}{195}a^{6}q^{3}\frac{\sigma }{m}-\frac{352}{105}a^{6}q\frac{\sigma }{m}-\frac{27073}{6435}a^{4}m^{2}q^{6}+\frac{1340}{429}a^{4}m^{2}q^{4}\\ & \;-\frac{54947}{6435}a^{4}m^{2}q^{2}-\frac{2986}{15015}a^{4}mq^{7}\sigma -\frac{7496}{1287}a^{4}mq^{5}\sigma +\frac{127454}{6435}a^{4}mq^{3}\sigma \\ & \;-\frac{3368}{693}a^{4}mq\sigma +\frac{27073}{6435}a^{2}m^{4}q^{6}+\frac{1217}{117}a^{2}m^{4}q^{4}-\frac{14206}{2145}a^{2}m^{4}q^{2}\\ & \;+\frac{2986}{15015}a^{2}m^{3}q^{7}\sigma +\frac{75908}{6435}a^{2}m^{3}q^{5}\sigma -\frac{116924}{6435}a^{2}m^{3}q^{3}\sigma +\frac{256}{1155}a^{2}m^{3}q\sigma \\ & \;-\frac{27073}{19305}m^{6}q^{6}-\frac{30794}{3861}m^{6}q^{4}+\frac{5048}{19305}m^{6}q^{2}-\frac{2986}{45045}m^{5}q^{7}\sigma -\frac{12704}{2145}m^{5}q^{5}\sigma \\ & \;-\frac{4544}{2145}m^{5}q^{3}\sigma -\frac{32}{3465}m^{5}q\sigma )\\ M_{8} & =M(m^{8}+9m^{7}q\sigma +28m^{6}q^{2}\sigma^{2}-4m^{6}\sigma^{2}+\frac{3812}{99}m^{5}q^{3}\sigma^{3}-\frac{3044}{99}m^{5}q\sigma^{3}\\ & \;+\frac{938}{33}m^{4}q^{4}\sigma^{4}-\frac{2480}{33}m^{4}q^{2}\sigma^{4}+6m^{4}\sigma^{4}+\frac{32938}{2145}m^{3}q^{5}\sigma^{5}-\frac{30896}{429}m^{3}q^{3}\sigma^{5}\\ & \;+\frac{27154}{715}m^{3}q\sigma^{5}+\frac{821452}{135135}m^{2}q^{6}\sigma^{6}-\frac{932884}{27027}m^{2}q^{4}\sigma^{6}+\frac{8883508}{135135}m^{2}q^{2}\sigma^{6}\\ & \;-4m^{2}\sigma^{6}+\frac{24844}{10395}mq^{7}\sigma^{7}-\frac{1630604}{135135}mq^{5}\sigma^{7}+\frac{4516228}{135135}mq^{3}\sigma^{7}\\ & \;-\frac{887132}{45045}mq\sigma^{7}+\frac{257}{385}q^{8}\sigma^{8}-\frac{96568}{45045}q^{6}\sigma^{8}+\frac{100322}{15015}q^{4}\sigma^{8}\\ & \;-\frac{836312}{45045}q^{2}\sigma^{8}+\sigma^{8}+\frac{257}{3465}q^{9}\frac{\sigma^{9}}{m}-\frac{2152}{45045}q^{7}\frac{\sigma^{9}}{m}-\frac{12874}{45045}q^{5}\frac{\sigma^{9}}{m}\\ & \;-\frac{5128}{45045}q^{3}\frac{\sigma^{9}}{m}+\frac{1091}{315}q\frac{\sigma^{9}}{m}) \end{array}$$

3.11 $$\begin{array}{rl} S_{9} & =S_{0}(\frac{177511}{255255}a^{8}q^{8}-\frac{26024}{12155}a^{8}q^{6}+\frac{1912994}{255255}a^{8}q^{4}-\frac{1918792}{85085}a^{8}q^{2}+a^{8}\\ & \;-\frac{20486}{6891885}a^{8}q^{9}\frac{\sigma }{m}+\frac{59144}{328185}a^{8}q^{7}\frac{\sigma }{m}-\frac{149956}{208845}a^{8}q^{5}\frac{\sigma }{m}+\frac{155192}{530145}a^{8}q^{3}\frac{\sigma }{m}+\frac{1126}{315}a^{8}q\frac{\sigma }{m}\\ & \;-\frac{710044}{255255}a^{6}m^{2}q^{8}-\frac{1751252}{255255}a^{6}m^{2}q^{6}+\frac{2530868}{85085}a^{6}m^{2}q^{4}+\frac{97892}{12155}a^{6}m^{2}q^{2}\\ & \;+\frac{81944}{6891885}a^{6}mq^{9}\sigma -\frac{13208144}{2297295}a^{6}mq^{7}\sigma +\frac{53663312}{2297295}a^{6}mq^{5}\sigma \\ & \;-\frac{346745888}{6891885}a^{6}mq^{3}\sigma +\frac{61448}{9009}a^{6}mq\sigma +\frac{355022}{85085}a^{4}m^{4}q^{8}+\frac{568852}{17017}a^{4}m^{4}q^{6}\\ & \;-\frac{6788302}{85085}a^{4}m^{4}q^{4}+\frac{265388}{17017}a^{4}m^{4}q^{2}-\frac{40972}{2297295}a^{4}m^{3}q^{9}\sigma +\frac{12380128}{765765}a^{4}m^{3}q^{7}\sigma \\ & \;-\frac{4893500}{153153}a^{4}m^{3}q^{5}\sigma +\frac{8150456}{208845}a^{4}m^{3}q^{3}\sigma -\frac{6416}{15015}a^{4}m^{3}q\sigma -\frac{710044}{255255}a^{2}m^{6}q^{8}\\ & \;-\frac{3208596}{85085}a^{2}m^{6}q^{6}+\frac{1471432}{36465}a^{2}m^{6}q^{4}-\frac{8864}{7735}a^{2}m^{6}q^{2}+\frac{81944}{6891885}a^{2}m^{5}q^{9}\sigma \\ & \;-\frac{36312368}{2297295}a^{2}m^{5}q^{7}\sigma -\frac{7586872}{2297295}a^{2}m^{5}q^{5}\sigma +\frac{79889104}{6891885}a^{2}m^{5}q^{3}\sigma +\frac{1504}{45045}a^{2}m^{5}q\sigma \\ & \;+\frac{177511}{255255}m^{8}q^{8}+\frac{260828}{19635}m^{8}q^{6}+\frac{16948}{7735}m^{8}q^{4}+\frac{4112}{85085}m^{8}q^{2}-\frac{20486}{6891885}m^{7}q^{9}\sigma \\ & \;+\frac{2393224}{459459}m^{7}q^{7}\sigma +\frac{4139368}{328185}m^{7}q^{5}\sigma -\frac{825152}{1378377}m^{7}q^{3}\sigma -\frac{64}{45045}m^{7}q\sigma )\\ M_{10} & =M(-m^{10}-11m^{9}q\sigma -45m^{8}q^{2}\sigma^{2}+5m^{8}\sigma^{2}-\frac{38075}{429}m^{7}q^{3}\sigma^{3}+\frac{20645}{429}m^{7}q\sigma^{3}\\ & \;-\frac{41510}{429}m^{6}q^{4}\sigma^{4}+\frac{70040}{429}m^{6}q^{2}\sigma^{4}-10m^{6}\sigma^{4}-\frac{150698}{2145}m^{5}q^{5}\sigma^{5}+\frac{35528}{143}m^{5}q^{3}\sigma^{5}\\ & \;-\frac{176062}{2145}m^{5}q\sigma^{5}-\frac{27070}{693}m^{4}q^{6}\sigma^{6}+\frac{1752710}{9009}m^{4}q^{4}\sigma^{6}-\frac{1966522}{9009}m^{4}q^{2}\sigma^{6}\\ & \;+10m^{4}\sigma^{6}-\frac{884582}{51051}m^{3}q^{7}\sigma^{7}+\frac{25565266}{255255}m^{3}q^{5}\sigma^{7}-\frac{11654186}{51051}m^{3}q^{3}\sigma^{7}\\ & \;+\frac{17219134}{255255}m^{3}q\sigma^{7}-\frac{3200647}{459459}m^{2}q^{8}\sigma^{8}+\frac{16620592}{459459}m^{2}q^{6}\sigma^{8}-\frac{48051098}{459459}m^{2}q^{4}\sigma^{8}\\ & \;+\frac{58197248}{459459}m^{2}q^{2}\sigma^{8}-5m^{2}\sigma^{8}-\frac{66272933}{26189163}mq^{9}\sigma^{9}+\frac{12124624}{1247103}mq^{7}\sigma^{9}\\ & \;-\frac{139964914}{4849845}mq^{5}\sigma^{9}+\frac{14896480}{220077}mq^{3}\sigma^{9}-\frac{380725673}{14549535}mq\sigma^{9}-\frac{443699}{793611}q^{10}\sigma^{10}\\ & \;+\frac{83879}{73359}q^{8}\sigma^{10}-\frac{49852798}{43648605}q^{6}\sigma^{10}+\frac{62188166}{8729721}q^{4}\sigma^{10}-\frac{387595759}{14549535}q^{2}\sigma^{10}\\ & \;+\sigma^{10}-\frac{443699}{8729721}q^{11}\frac{\sigma^{11}}{m}+\frac{522479}{26189163}q^{9}\frac{\sigma^{11}}{m}+\frac{19539686}{43648605}q^{7}\frac{\sigma^{11}}{m}-\frac{3438746}{2567565}q^{5}\frac{\sigma^{11}}{m}\\ & \;+\frac{110793707}{130945815}q^{3}\frac{\sigma^{11}}{m}+\frac{12701}{3465}q\frac{\sigma^{11}}{m}). \end{array}$$

The MN and the QM metrics might be useful to represent the space–time of a real non-collapsed body due to their large freedom in choosing the multipole moments of the exterior gravitational field. Nevertheless, the higher-order spin moments cannot be chosen freely but are totally determined by the set of metric parameters. The *q*-metric may be useful to represent the space–time of deformed objects, but again the higher order spin moments cannot be chosen freely. It is interesting to analyse some particular cases from the multipole structures for these space–times. For an extreme black hole, all these metrics are isometrics with relativistic multipoles of the form $M_{2n}=\pm Mm^{2n}$ and $S_{2n+1}=\pm Mm^{2n+1}$ as expected. For neutron stars, the forms of the first five multipole moments are [36–38]

3.12 $$\begin{array}{rl} M_{0} & =M,\\ S_{1} & =S=J=Mac,\\ M_{2} & =-\alpha \frac{S^{2}}{M},\\ S_{3} & =-\beta \frac{S^{3}}{M^{2}}\\ \;\mathrm{and}\;M_{4} & =\gamma \frac{S^{4}}{M^{3}}, \end{array}\}$$

where *α*,  *β*,   *γ* are parameters. For the MN metric, if we set a→−a, *Q* = (1 − *α*)*S*^2^/*M* and

$$k\alpha_{4}\beta^{4}Mm^{3}=\frac{S^{4}}{M^{3}}\left((4\alpha -\gamma -3)-\frac{8}{7}(1-\alpha )m^{2}a^{2}\right)$$

in (3.3), then the values (3.12) are reproduced, except for $S_{3}=(4\alpha -3)S^{3}/M^{2}$.

In the case of the QM metric, if one sets *β*^3^*Q* = (1 − *α*)*S*^2^/*M* and

$$\frac{8}{315}\beta^{5}q_{4}Mm^{4}=\frac{S^{4}}{M^{3}}\left(\gamma -1-\frac{20}{7}(1-\alpha )\frac{a^{2}}{\beta^{2}m^{2}}+\frac{2}{7}(1-\alpha )\frac{1}{\beta^{2}}\left(\frac{m^{2}}{a^{2}}+9\right)\right)$$

in (3.7), we get the values (3.12), except for $S_{3}=(1-2\alpha )S^{3}/M^{2}$. Finally, for the *q*-metric (see (3.9)), it is not possible to construct all values (3.12).

In the next section, we compare the static multipoles of the MN metric derived from (3.3) and the ER multipole moments deduced from (3.7) with those ones of different static metrics.

## 4. Comparisons with static metrics

For a comparison with other static metrics, we consider the Gutsunayev–Manko (GM) [39], the Hernández–Martín space–times (HM I and II) [40], and the static *q*-metric [17,34]. These metrics are static solutions of the EFE and might be related to the external gravitational field of a body with mass and quadrupole moment. The differences of the metrics are in the fields *ψ* and *γ*. To obtain the multipole moments of these metrics, the FHP procedure was also employed. In the case of the generalized ER metric, we have from (3.7) the following mass multipoles:

$$\begin{array}{rl} M_{0} & =M,\\ M_{2} & =Q,\\ M_{4} & =\frac{2}{21}(-3Q+60q_{4}Mm^{2})m^{2},\\ M_{6} & =-\frac{8}{3003}(13(1+3q_{2})Q+30(13q_{4}-15q_{6})Mm^{2})m^{4},\\ M_{8} & =-\frac{16}{765765}((255-629q_{2}+238q_{2}^{2}+1921q_{4})Q\\ & \;+30(255q_{4}+255q_{6}-420q_{8})Mm^{2})m^{6}\\ \;\mathrm{and}\;M_{10} & =-\frac{32}{2909907}((84-481q_{2}-1124q_{2}^{2}-187q_{4}+261q_{2}q_{4}+849q_{6})Q\\ & \;+30(84q_{4}+294q_{4}^{2}+84q_{6}+84q_{8}-180q_{10})Mm^{2})m^{8}. \end{array}$$

In the case of the MN metric if *α* = *a* = 0 , we have

4.1 $$\begin{array}{rl} M_{0} & =M=k,\\ M_{2} & =Q,\\ M_{4} & =-\frac{8}{7}Qm^{2}-\alpha_{4}Mm^{4},\\ M_{6} & =Qm\left(\frac{19}{33}m^{3}+\frac{60}{77}k^{3}\alpha_{2}\right)+\frac{1}{11}Mm^{6}(17\alpha_{4}-11\alpha_{6}),\\ M_{8} & =-Q\left(\frac{34}{143}m^{6}+k^{3}\alpha_{2}\left(\frac{4364}{3003}m^{3}+\frac{40}{143}k^{3}\alpha_{2}\right)\right)\\ & \;-\alpha_{4}Mm^{5}\left(\frac{16}{13}m^{3}+\frac{226}{143}k^{3}\alpha_{2}\right)+Mm^{8}(2\alpha_{6}-\alpha_{8})\\ \;\mathrm{and}\;M_{10} & =Qm^{2}\left(\frac{371}{4199}m^{6}+k^{3}\alpha_{2}\left(\frac{182530}{138567}m^{3}+k^{3}\alpha_{2}\frac{459700}{323323}\right)\right)\\ & \;+\frac{1}{46189}\alpha_{4}Mm^{4}\left(34034m^{6}+k^{3}\alpha_{2}\left(167030m^{3}+39150k^{3}\alpha_{2}\right)\right)\\ & \;-\frac{30870}{46189}\alpha_{4}^{2}Mm^{10}-\frac{1}{323}\alpha_{6}Mm^{7}(686m^{3}+566k^{3}\alpha_{2})\\ & \;+\frac{1}{19}Mm^{10}(47\alpha_{8}-19\alpha_{10}). \end{array}\}$$

For the GM metric [39] and the HM [40], one uses the following Ernst potential:

4.2 $$\xi =\frac{x\;\tanh\;\psi +1}{x+\;\tanh\;\psi }.$$

The field *ψ* for the GM is given by

4.3 $$\psi =q\frac{x}{{\left(x^{2}-y^{2}\right)}^{3}}(x^{2}-3x^{2}y^{2}+3y^{2}-y^{4})$$

and the lowest six mass multipoles read

$$\begin{array}{rl} M_{0} & =M,\\ M_{2} & =Q,\\ M_{4} & =\frac{6}{7}Qm^{2},\\ M_{6} & =\frac{8}{231}Qm^{4}\left(14-45q\right),\\ M_{8} & =\frac{8}{3003}Qm^{6}\left(84-1282q-420q^{2}\right)\\ \;\mathrm{and}\;M_{10} & =\frac{32}{3927}Qm^{8}\left(\frac{2772}{247}-\frac{1343804}{2717}q-\frac{2550}{19}q^{2}\right), \end{array}$$

where *Q* = 2*qMm*^2^.

For static metrics, Hernández & Martín [40] have shown that the Weyl moments *a*_*l*_, defined by

$$\psi =\sum_{l=0}^{\infty }\frac{a_{l}}{r^{l+1}}P_{l}(\cos\theta )$$

((*r*,  *θ*) are spherical Weyl coordinates), can be found if the relativistic multipole moments are given and vice versa. It is easy to see that for a stationary metric, one can invert the FHP formulae to find the metric coefficients. We have written a REDUCE program (available upon request) to invert the FHP formulae. Thus, the metric structure can be determined in principle from the relativistic multipole moments. There are two HM solutions. In case of the first HM metric, the field *ψ* is given by

4.4 $$\psi =\frac{5}{8}q\left[\frac{1}{4}((3x^{2}-1)(3y^{2}-1)-4)\ln\left[\frac{x-1}{x+1}\right]-\frac{2x}{\left(x^{2}-y^{2}\right)}+\frac{3}{2}x(3y^{2}-1)\right]$$

and the lowest mass multipoles are given by

$$\begin{array}{rl} M_{0} & =M,\\ M_{2} & =Q,\\ M_{4} & =0,\\ M_{6} & =-\frac{60}{77}qQm^{4},\\ M_{8} & =-\frac{4}{3003}qQm^{6}(265+210q)\\ \;\mathrm{and}\;\;M_{10} & =\frac{4}{3927}qQm^{8}\left(-\frac{34790}{247}+\frac{769125}{1729}q\right), \end{array}$$

where *Q* = *qMm*^2^. For the second HM metric, the field *ψ* is given by (4.4) plus a second term with the parameter *q*^2^ (see [34,40]). The lowest six mass multipoles are given by

$$\begin{array}{rl} M_{0} & =M,\\ M_{2} & =Q,\\ M_{4} & =0,\\ M_{6} & =0,\\ M_{8} & =-\frac{40}{143}q^{2}Qm^{6}\\ \;\mathrm{and}\;M_{10} & =-\frac{42140}{46189}q^{2}Qm^{8}. \end{array}$$

Finally from (2.26), we get the following mass multipoles for the *q*-metric:

$$\begin{array}{rl} M_{0} & =\delta M,\\ M_{2} & =Q=\frac{1}{3}\delta Mm^{2}(1-\delta^{2}),\\ M_{4} & =\delta Mm^{4}\left(\frac{19}{105}\delta^{4}-\frac{8}{21}\delta^{2}+\frac{1}{5}\right),\\ M_{6} & =\delta Mm^{6}\left(-\frac{389}{3465}\delta^{6}+\frac{23}{63}\delta^{4}-\frac{457}{1155}\delta^{2}+\frac{1}{7}\right),\\ M_{8} & =\delta Mm^{8}\left(\frac{257}{3465}\delta^{8}-\frac{44312}{135135}\delta^{6}+\frac{73522}{135135}\delta^{4}-\frac{54248}{135135}\delta^{2}+\frac{1}{9}\right)\\ \;\mathrm{and}\;M_{10} & =\delta Mm^{10}\left(-\frac{443699}{8729721}\delta^{10}+\frac{17389}{61047}\delta^{8}-\frac{27905594}{43648605}\delta^{6}+\frac{6270226}{8729721}\delta^{4}-\frac{5876077}{14549535}\delta^{2}+\frac{1}{11}\right), \end{array}$$

where *δ* = 1 + *q*.

The multipole structures of the ER, the static MN, the GM, the HM (I and II) and *q*-metrics are different and the space–times are not isometric to each other. For modelling the exterior metric of a realistic static body the generalized ER or QM metric and MN metric with their infinitely many independent mass moments *q*_*n*_ and *α*_*n*_ that can be chosen freely might be employed.

## 5. Conclusion

In this contribution, we gave a brief review of the QM space–time, and the MN metric. These metrics contain an infinite set of multipole moments that in principle can be chosen freely. The rotating *q*-metric was also introduced. This one has only three parameters: mass, rotation parameter and quadrupole. Therefore, it may be useful to represent deformed objects. The relativistic multipole moments for these metrics were derived using the corresponding Ernst function and the FHP formalism. The Ernst function takes a simple form for even values of *α*_*n*_ and *q*_*n*_ and then it allows us to deduce the GH moments. In principle, it is possible to extent the REDUCE program to include zonal harmonics with odd values of *n* (that violate equatorial symmetry).

Although the MN and the QM metrics have a large set of multipole moment parameters for the exterior gravitational field, their higher-order spin moments cannot be chosen freely, because they are totally determined by the set of metric parameters. The GH moments for the generalized ER metric and those of the static MN metric were compared with those of other metrics, namely the GM, HM (I and II), and *q*-metrics. All compared space–times are not isometric to each other. The generalized ER or QM and the MN metrics are more appropriate to describe the exterior gravitational field of a real static object due to their more general multipole structures.

## Data accessibility

This article has no additional data.

## References

[1] Ernst FJ 1968 New formulation of the axially symmetric gravitational field problem. Phys. Rev. 167, 1175–1178. (10.1103/PhysRev.167.1175)

[2] Ernst FJ 1968 New formulation of the axially symmetric gravitational field problem II. Phys. Rev. 168, 1415–1417. (10.1103/PhysRev.168.1415)

[3] Hoenselaers C, Kinnersley W, Xanthopoulos BC 1979 Symmetries of the stationary Einstein-Maxwell equations. VI. Transformations which generate asymptotically flat spacetimes with arbitrary multipole moments. J. Math. Phys. 20, 2530–2536. (10.1063/1.523580))

[4] Belinsky A, Zakharov VE 1978 Integration of the Einstein equations by means of the inverse scattering problem technique and construction of exact soliton solutions. Soviet Physics (JETP) 48, 985–994.

[5] Belinsky A, Zakharov VE 1979 Stationary gravitational solitons with axial symmetry. Soviet Physics (JETP) 50, 1–9.

[6] Quevedo H, Mashhoon B 1985 Exterior gravitational field of a rotating deformed mass. Phys. Lett. 109A, 13–18. (10.1016/0375-9601(85)90381-0)

[7] Quevedo H, Mashhoon B 1990 Exterior gravitational field of a charged rotating mass with arbitrary quadrupole moment. Phys. Lett. 148A, 149–153. (10.1002/10.1016/0375-9601(90)90770-O)

[8] Quevedo H 1990 Multipole moments in general relativity—static and stationary vacuum solutions. Fortschritte der Physik 38, 733–840. (10.1002/prop.2190381002)

[9] Carmeli M 2001 Classical fields: general relativity and gauge theory. Singapore: World Scientific Publishing.

[10] Boshkayev K, Quevedo H, Ruffini R 2012 Gravitational field of compact objects in general relativity. Phys. Rev. D 86, 064043 (10.1103/PhysRevD.86.064043)

[11] Yamazaki M 1978 On the Kerr-Tomimatsu-Sato family of solutions with nonintegral distortion parameter. J. Math. Phys. 19, 1847–1849. (10.1063/1.523925)

[12] Yamazaki M 1981 On the Hoenselaers-Kinnersley- Xanthopoulos spinning mass fields. J. Math. Phys. 22, 133–135. (10.1063/1.524744))

[13] Cosgrove CM 1980 Relationships between the group theoretic and soliton theoretic techniques for generating stationary axisymmetric gravitational solutions. J. Math. Phys. 21, 2417–2447. (10.1063/1.524680)

[14] Dietz W, Hoenselaers C 1982 A new class of bipolar vacuum gravitational fields. Proc. R. Soc. Lond. A 382, 221–229. (10.1098/rspa.1982.0098)

[15] Manko VS, Novikov ID 1992 Generalizations of the Kerr and Kerr-Newman metrics possessing an arbitrary set of mass-multipole moments. Class. Quatum Gravity 9, 2477–2487. (10.1088/0264-9381/9/11/013)

[16] Toktarbay S, Quevedo H 2014 A stationary *q*-metric. Gravit. Cosmol. 20, 252–254. (10.1134/S0202289314040136)

[17] Quevedo H 2011 Mass quadrupole as a source of naked singularities. Int. J. Mod. Phys. D 20, 1779–1787. (10.1142/S0218271811019852)

[18] Quevedo H, Toktarbay S, Yerlan A 2012 Quadrupolar gravitational fields described by the *q*-metric. Int. J. Math. Phys. 3, 133–139.

[19] Panhans M, Soffel M 2014 Gravito-magnetism of an extended celestial body. Class. Quantum Gravity 31, 245012 (10.1088/0264-9381/31/24/245012)

[20] Teyssandier P 1978 Rotating stratified ellipsoids of revolution and their effects on the dragging of inertial frames. Phys. Rev. D 18, 1037–1046. (10.1103/PhysRevD.18.1037)

[21] Geroch R 1970 Multipole moments. II. Curved space. J. Math. Phys. 11, 2580–2588. (10.1063/1.1665427)

[22] Hansen RO 1974 Multipole moments of stationary space-times. J. Math. Phys. 15, 46–52. (10.1063/1.1666501)

[23] Simon W, Beig R 1983 The multipole structure of stationary space-times. J. Math. Phys. 24, 1163–1171. (10.1063/1.525846)

[24] Thorne KS 1980 Multipole expansions of gravitational radiation. Rev. Mod. Phys. 52, 299–340. (10.1103/RevModPhys.52.299)

[25] Gürsel Y 1983 Multipole moments for stationary systems: the equivalence of the Geroch-Hansen formulation and the Thorne formulation. Gen. Relativ. Gravit. 15, 737–754. (10.1007/BF01031881)

[26] Fodor G, Hoenselaers C, Perjés Z 1989 Multipole moments of axisymmetric systems in relativity. J. Math. Phys. 30, 2252–2257. (10.1063/1.528551)

[27] Hoenselaers C, Perjés Z 1990 Multipole moments of axisymmetric electrovacuum spacetimes. Class. Quantum Gravity 7, 1819–1825. (10.1088/0264-9381/7/10/012)

[28] Ryan FD 1995 Gravitational waves from the inspiral of a compact object into a massive, axisymmetric body with arbitrary multipole moments. Phys. Rev. D 52, 5707–5718. (10.1103/PhysRevD.52.5707) 10019103

[29] Quevedo H 2011 Exterior and interior metrics with quadrupole moment. Gen. Relativ. Gravit. 43, 1141–1152. (10.1007/s10714-010-0940-5)

[30] Quevedo H 1986 Class of stationary axisymmetric solutions of Einstein's equations in empty space. Phys. Rev. D 33, 324–327. (10.1103/PhysRevD.33.324) 9956622

[31] Quevedo H, Mashhoon B 1991 Generalization of Kerr spacetime. Phys. Rev. D 43, 3902–3906. (10.1103/PhysRevD.43.3902) 10013356

[32] Quevedo H 1989 General static axisymmetric solution of Einstein's vacuum field equations in prolate spheroidal coordinates. Phys. Rev. D 39, 2904–2911. (10.1103/PhysRevD.39.2904) 9959519

[33] Hearn AC 2004 *REDUCE user's and contributed packages manual*. Berlin, Germany: Konrad-Zuse-Zentrum für Informationstechnik.

[34] Frutos-Alfaro F, Quevedo H, Sanchez P 2018 Comparison of vacuum static quadrupolar metrics. R. Soc. open sci. 5, 170826 (10.1098/rsos.170826) 29892343 PMC5990784

[35] Filter RJ 2008 Multipolmomente axialsymmetrisch stationärer Raumzeiten und die Quadrupol- Vermutung. Diploma thesis (in German), Friedrich-Schiller-Universität. Jena, Germany. http://robertfilter.net/files/Diplomarbeit_RobertFilter.pdf.

[36] Pappas G, Apostolatos TA 2014 Effectively universal behavior of rotating neutron stars in general relativity makes them even simpler than their Newtonian counterparts. Phys. Rev. Lett. 112, 121101 (10.1103/PhysRevLett.112.121101) 24724643

[37] Yagi K, Kyutoku K, Pappas G, Yunes N, Apostolatos TA 2014 Effective no-hair relations for neutron stars and quark stars: relativistic results. Phys. Rev. D 89, 124013 (10.1103/PhysRevD.89.124013)

[38] Pappas G 2017 An accurate metric for the spacetime around neutron stars. Mon. Not. R. Astronom. Soc. 466, 4381–4394. (10.1093/mnras/stx019)

[39] Gutsunayev TsI, Manko VS 1985 On the gravitational field of a mass possessing a multipole moment. Gen. Relativ. Gravit. 17, 1025–1027. (10.1007/BF00774205)

[40] Hernández-Pastora JL, Martí J 1994 Monopole-quadrupole static axisymmetric solutions of Einstein field equations. Gen. Relativ. Gravit. 26, 877–907. (10.1007/BF02107146)

